# Under-reporting of foetal alcohol spectrum disorders: an analysis of hospital episode statistics

**DOI:** 10.1186/1471-2431-11-14

**Published:** 2011-02-08

**Authors:** Michela Morleo, Kerry Woolfall, Dan Dedman, Raja Mukherjee, Mark A Bellis, Penny A Cook

**Affiliations:** 1 Centre for Public Health, Liverpool John Moores University, Henry Cotton Campus (third floor), 15-21 Webster Street, Liverpool L3 2ET, UK; 2 MRC North West Hub for Trials Methodology Research, Department of Mental Health and Well-being, Institute of Psychology, Health and Society, University of Liverpool, Whelan Building, Brownlow Hill, Liverpool L69 3GB, UK; 3 School of Public Health and Clinical Sciences, University of Central Lancashire, Preston PR1 2HE, UK; 4 FASD Specialist Behavioural Clinic, Surrey and Border Partnership NHS Foundation Trust, 116-118 Station Road East, Oxted, Surrey, RH8 0QA UK

## Abstract

**Background:**

Internationally, 0.97 per 1,000 live births are affected by foetal alcohol syndrome (FAS). However, prevalence intelligence has been limited in the UK, hindering the development of appropriate services. This analysis compares hospital admissions over time, between regions and with alcohol-related admissions for adult females to assess whether established patterns (such as the North experiencing elevated harms) can be identified.

**Methods:**

A retrospective analysis of hospital admissions data (April 2002 to March 2008) for foetal alcohol spectrum disorder (FASD)-related conditions: foetal alcohol syndrome (dysmorphic) (n = 457); foetus and newborn affected by maternal use of alcohol (n = 157); maternal care for (suspected) damage to foetus from alcohol (n = 285); and 322,161 women admitted due to alcohol-related conditions.

**Results:**

Whilst the rate of admission for alcohol-related conditions in women aged 15-44 years increased significantly by 41% between 2002/03 and 2007/08 (p < 0.0001), no such increases were seen in the numbers of FASD-related conditions (all p < 0.05). Established regional rates of admission for alcohol-related conditions in women aged 15-44 years old were not associated with admission for FASD-related conditions.

**Conclusions:**

It would be expected that the North West and North East regions, known to have higher levels of alcohol harm would have higher levels of FASD-related conditions. However, this was not reflected in the incidence of such conditions, suggesting under-reporting. With incomplete datasets, intelligence systems are severely limited, hampering efforts to develop targeted interventions. Improvements to intelligence systems, practitioner awareness and screening are essential in tackling this.

## Background

Worldwide estimates suggest that 0.97 per 1,000 live births are affected by foetal alcohol syndrome (FAS) [1], representing a significant cost to health services, with each affected baby estimated to cost a mean of $2,842 annually [2]. However, long-term costs may be much higher; FAS is associated with psychiatric problems, drug and alcohol addiction, memory and attention deficits [3,4], thus affecting a range of services including criminal justice, education as well as impacting on the family and local community. In the United Kingdom (UK), prevalence data are limited. Five UK studies contributed to the worldwide estimate; none identified FAS but they were restricted in sample size and geographic representation [1,5-10]. Subsequently, an analysis of Hospital Episode Statistics (HES) reported 128 cases in 2002/03 in England [3]. However, alcohol-related harm has since escalated [11,12].

Rates of alcohol related hospital admission for alcohol-related liver disease increased by over 100% between 1989/90 and 2002/03 in England and Wales [12]. Women are particularly at risk of alcohol-related harm due to biological and social factors [13-15]. For example, women are more likely to pre-load than men, a behaviour associated with higher risk of alcohol-related harm [15,16]. Such risk factors are of particular importance in the early stages of pregnancy, when the risks may be higher (the National Institute for Health and Clinical Excellence advises abstinence in the first three months) [17] and women are less likely to be aware of their pregnancy status. Levels of consumption are significant in young women, with one study in North West England showing that female nightlife users reported an average consumption of 16.5 units in one night, five times the recommended daily maximum of three units [18]. Even low levels of alcohol consumption, especially if consumed regularly, may affect health. Of women admitted to hospital for unspecified liver cirrhosis in England in 2005/06, 44.4% were estimated to drink from 0.1 to 2.4 units per day [19]. With no clear guidance on alcohol consumption in pregnancy and no established dose related threshold to distinguish between safe and harmful levels of consumption [3,20], pregnant women can be confused by advice around alcohol consumption [21], increasing the potential risk for alcohol misuse and the development of conditions such as FAS. However, without adequate monitoring of prevalence of these conditions, it is no possible to establish the true impact of consumption on unborn children in the UK.

We explore the reporting on foetal alcohol spectrum disorder (FASD) related disorder in England using HES. Hospital admissions were compared over time, between regions and with alcohol-related hospital admissions for adult females. We assess whether known geographical patterns in alcohol related harms (for example, whereby the North experiences elevated levels of alcohol-attributable hospital admissions and incapacity benefits claimants for alcoholism)[11] are reflected in FAS-related admission.

## Methods

HES is a data warehouse containing details of all admissions to National Health Service (NHS) hospitals in England, as well as NHS funded inpatient care provided by independent treatment centres. Each record relates to an episode of care under a single consultant or medical team. Up to 20 diagnoses can be recorded for each episode (increasing from 14 in 2007/08). Diagnoses are coded using the International Classification of Diseases, 10^th ^revision (ICD10). We used 4-digit ICD10 codes to identify inpatient episodes: O35.4, maternal care for (suspected) damage to foetus from alcohol; P04.3, foetus and newborn affected by maternal use of alcohol; and Q86.0, FAS (dysmorphic). Using HES for the UK financial years (April to March) 2002/03 to 2007/08, we identified episodes of care where any one of these diagnoses was recorded. Details extracted included: age at start of episode; sex; Government Office Region (GOR) of residence (from nine across England); and person identifier (HESID). The person identifier is a derived variable that links episodes of care to the same individual (based on NHS number and other identifiers), and which can be used to exclude repeat admissions for the same individual. Using the person identifier, we estimated the number of individuals who were admitted to hospital with each condition in each financial year. This measure combines incident and prevalent cases arising in a 12 month period, and allowed us to assess regional and temporal reporting trends in admission rates which were free from potential distortions arising from multiple hospital episodes in the same individual.

For admissions for foetus and newborn affected by maternal use of alcohol (ICD10 P04.3), we calculated rates using the number of live births in the relevant year as the denominator [22]. Live births were also used as the denominator for admission rates for maternal care for (suspected) damage to foetus from alcohol (O35.4). For FAS (Q86.0), since a diagnosis is often not made until later in childhood [3,23], we calculated hospital admission rates for children aged up to 14 years, and used mid-year population estimates for children aged up to 14 years as denominators. Here, we excluded 50 episodes involving patients aged 15 or over.

In addition, we used HES to estimate the number of women aged 15-44 years who were admitted to hospital with an alcohol-related diagnosis from 2002/03 to 2007/08. The methods used for this are described in detail elsewhere [19], but involve identifying numbers of individuals admitted with a range of alcohol-related conditions. For each condition, an alcohol-attributable fraction (AAF) was applied, which represents the proportion of cases where all alcohol cases are alcohol-related by definition and the AAF equals 1 (or 100%). It also includes conditions where alcohol is a contributory factor in only a proportion of cases, for example road traffic accidents (AAF: 0.09-0.21 for females aged 15-44 years), and cancers of the lip, oral cavity and pharynx (AAF: 0.35-0.40). Rates were calculated using mid-year population estimates for women aged 15-44 years as denominators. The rates provide a proxy measure of overall levels of alcohol-related harm in women of childbearing age, and can be compared with admission rates for FASD and related conditions in children.

HES also contains data for NHS outpatient appointments, and we examined the reporting of FASD-related conditions here for 2003/04 to 2007/08 [24]. However, because it is not mandatory for providers to code diagnoses on outpatient records, the completeness of the diagnosis fields is very low. Of 54 million outpatient appointments recorded in 2007/08, less than 3% of outpatient episodes have a valid diagnosis field [25], and we therefore predicted that these data would be unsuitable for assessing regional or temporal trends in FASD-related conditions. In fact, no cases were identified from the outpatient records and so no analysis was possible.

Trends in reporting over time and between regions were assessed using Poisson regression models, with likelihood ratio tests used to assess temporal and regional variation. We examined associations between regional admission rates for FASD-related conditions and alcohol-related harm in women of childbearing age using Pearson's correlation coefficient. Analyses were performed using Stata 10. Ethical approval was not required [26], as secondary analysis of HES data can be used to identify public health issues and for general medical research under existing protocols. All analyses performed complied with these regulations [27].

## Results

Between 2002/03 and 2007/08, there were 987 episodes with a diagnosis of FAS (ICD10 code Q86.0) involving 457 children aged under 15 years (Tables 1 and 2). Around 36% of children were aged under 1 at the time of admission (Table 2). The number of persons admitted increased substantially in 2006/07, relative to earlier years, and remained high in 2007/08. The overall trend was highly significant (p = 0.0001). Table 3 shows there were significant variations in regional rates (*p *< 0.0001 for heterogeneity). The North West had the highest rate of admissions at 1.67 (95%CIs: 1.39-1.99) per 100,000 population, while the lowest rates were seen in the North East (0.41 per 100,000; 95%CIs: 0.21-0.74).

**Table 1:** Trends in Hospital Episode Statistics for foetal alcohol spectrum disorder and related conditions, in residents of England 2002/03 to 2007/08.

	Q86.0: Foetal alcohol syndrome (dysmorphic), children aged under 15 years		P04.3: Foetus and newborn affected by maternal use of alcohol		O35.4: Maternal care for (suspected) damage to foetus from alcohol	
	
Financial year**	Persons (episodes)*		Persons (episodes)*		Persons (episodes)*	
	*n*	trend***	*n*	trend***	*n*	trend***
2002/03	71 (114)	Χ2(1 d.f.) = 15.48	32 (43)	Χ2 (1 d.f.) = 1.52	19 (21)	Χ2 (1 d.f.) = 1.95
2003/04	50 (84)	p = 0.0001	40 (42)	p = 0.22	19 (23)	p = 0.16
2004/05	69 (178)		55 (64)		29 (37)	
2005/06	73 (142)		62 (88)		33 (41)	
2006/07	96 (197)		45 (56)		26 (28)	
2007/08	98 (272)		51 (63)		31 (34)	
						
Total	457 (987)		285 (356)		157 (184)	

* The Hospital Episodes Statistics identification (HESID) field was used to link episodes relating to the same individual within a given year.

** The UK financial year runs from April to March.

*** A test of linear trend was obtained using likelihood ratio tests from Poisson regression models for rates based on number of individuals admitted.

**Table 2:** Hospital Episode Statistics for foetal alcohol spectrum disorder and related conditions, in residents of England 2002/03 to 2007/08 by person admitted*.

Patient characteristic	Q86.0: Foetal alcohol syndrome (dysmorphic), children aged under 15 years		P04.3: Foetus and newborn affected by maternal use of alcohol		O35.4: Maternal care for (suspected) damage to foetus from alcohol	
	
	*n*	*%*	*n*	*%*	*n*	*%*
Age group						
<1 month	104	22.8	278	97.5	0	0
2-11 months	62	13.6	4	1.4	0	0
1-4 years	155	33.9	1	0.4	0	0
5-14 years	136	29.8	0	0.0	0	0
15-24 years	N/A		0	0.0	39	24.8
25-44 years	N/A		1	0.4	118	75.2
Not known			1	0.4		
						
Sex						
Male	235	51.4	145	50.9	N/A	N/A
Female	222	48.6	139	48.8	157	100
Not known			1	0.4		
						
Total**	457	100	285	100	157	100

*The Hospital Episodes Statistics identification (HESID) field was used to exclude repeat episodes relating to the same individual within a given financial year (running April to March). **Percentages may not sum to 100% due to rounding.

**Table 3:** Reporting rates for foetal alcohol spectrum disorder and related conditions, and hospital admission rates for alcohol related conditions in women aged 15-44 years, England 2002/03 to 2007/08 (by person admitted*)

Government Office region of residence	Q86.0: Foetal alcohol syndrome (dysmorphic), children aged under 15 years			P04.3: Foetus and newborn affected by maternal use of alcohol			O35.4: Maternal care for (suspected) damage to foetus from alcohol			Women aged 15-44 years, admitted to hospital with alcohol related conditions**		
	
	*n*	Rate per 100,000 pop (95%CI)		*n*	Rate per 100,000 live births (95%CI)		*n*	Rate per 100,000 live births (95%CI)		*n*	Rate per 100,000 pop (95%CI)	
North East	11	0.41	(0.21-0.74)	3	1.8	(0.4-5.2)	4	2.4	(0.6-6.1)	23,561	757	(747-767)
North West	125	1.67	(1.39-1.99)	21	4.3	(2.7-6.6)	18	3.7	(2.2-5.9)	59,963	715	(709-721)
Yorkshire and Humber	30	0.54	(0.37-0.77)	19	5.3	(3.2-8.2)	12	3.3	(1.7-5.8)	35,990	571	(566-577)
East Midlands	32	0.69	(0.47-0.98)	17	5.8	(3.4-9.3)	21	7.2	(4.4-11)	27,029	516	(510-522)
West Midlands	32	0.54	(0.37-0.76)	9	2.3	(1.0-4.3)	25	6.3	(4.1-9.4)	34,186	529	(523-534)
East of England	46	0.76	(0.56-1.01)	20	5.2	(3.1-8.0)	11	2.8	(1.4-5.1)	26,637	402	(397-407)
London	68	0.83	(0.65-1.06)	17	2.5	(1.4-3.9)	12	1.7	(0.9-3.0)	40,894	375	(372-379)
South East	41	0.46	(0.33-0.63)	27	4.8	(3.1-6.9)	30	5.3	(3.6-7.5)	42,612	431	(427-435)
South West	31	0.60	(0.41-0.85)	17	5.4	(3.1-8.6)	23	7.2	(4.6-10.9)	29,840	517	(511-522)
England***	457	0.84	(0.76-0.92)	285	7.8	(6.9-8.7)	157	4.3	(3.6-5.0)	322,161	514	(512-516)

Correlation coefficient (*p-value*)****	0.26 (0.50)			-0.26 (0.50)			-0.08 (0.83)			--		

Test for heterogeneity	Χ^2^(8 d.f.) = 88.62, p < 0.0001			Χ^2^(8 d.f.) = 17.56, p = 0.025			Χ^2^(8 d.f.) = 33.43, p = 0.0001			Χ^2^(8 d.f.) = 17044, p < 0.0001		

*The Hospital Episodes Statistics identification (HESID) field was used to link episodes relating to the same individual within a given HES year (running April to March). Percentages may not sum to 100% due to rounding. ** See Jones et al. (2008) for details of attributable fractions applied. *** Figures for England include reports where region of residence was not recorded, and so the numbers provided are not the sum of the regional numbers. ****Pearson's correlation co-efficient was used to assess correlations between the prevalence of alcohol related hospital admission (for women aged 15-44) and FAS or related conditions across regions.

There were 356 episodes with a diagnosis of foetus and newborn affected by maternal use of alcohol (ICD10: P04.3) between 2002/03 and 2007/08, involving 285 individuals (Tables 1 and 2). Nearly all (99%) were aged under one year when admitted (Table 2). The number of persons admitted increased by 59%, from 32 in 2002/03 to 51 in 2007/08, but this trend was not statistically significant (p = 0.22). The rate of admissions varied significantly (p = 0.025) between regions, ranging from 1.8 per 100,000 live births in London (95%CIs: 0.4-5.2) to 5.8 per 100,000 live births in the East Midlands (95%CIs: 3.4-9.3; Table 3). However, the region of residence was missing for 47% of the patients, so that the overall rate for England was higher than any of the regional rates at 7.8 per 100,000 live births (95%CIs: 6.9-8.7).

There were 184 episodes of maternal care for (suspected) damage to the foetus from alcohol (ICD10: O35.4) in England, involving 157 individuals (Tables 1 and 2). A quarter were women aged 15-24 years and the remainder were over 24 years. The number of persons admitted between 2002/03 and 2007/08 increased by 63% from 19 in 2002/03 to 31 in 2007/08 but the overall trend was not statistically significant (p = 0.16). There was significant regional variation in rates (*p *= 0.0001), ranging from 1.7 per 100,000 live births in London (95%CIs: 0.9-3.0) to 7.2 in the East Midlands and South West (Table 3).

Admission rates for alcohol related conditions in women aged 15-44 increased by 41% between 2002/03 and 2007/08 from 418 to 591 per 100,000. This increase was highly significant (p < 0.0001). Admission rates ranged from 757 [747-767] per 100,000 women aged 15-44 years in the North East regions to 375 [372-379] per 100,000 in London (Table 3). There was no significant correlation between the regional admission rates for alcohol-related harm in women and admissions for the three alcohol-related diagnoses involving children or pregnant women.

## Discussion

It is extremely difficult to accurately estimate the prevalence of disorders such as FASD. There is uncertainty as to the level of maternal alcohol consumption that can cause FASD-related damage [4], and it can be difficult to obtain a valid understanding of consumption during pregnancy [28] as alcohol consumption amongst pregnant women is a highly sensitive area. In fact, experts in the United States of America suggest that the stigma attached to such disorders could reduce the likelihood of a diagnosis [28]. However, even without any associated stigma, diagnosis is difficult, not only due to the specialist training required [28,29] but also because affected individuals may have other diagnosable disorders or secondary disabilities [29], making it difficult to isolate FASD. Passive surveillance systems, such as HES used in this analysis, also present limitations. Intelligence may be restricted because systems rely on correct diagnosis by a large number of different medical practitioners [30]. Furthermore, trends in hospital data may be influenced by differential access and changes in service provision [31], as well as relying on individuals to have a reason to require hospital admission.

The HES data show an overall rate of hospital admission for FAS to be 0.84 per 100,000 population in England from 2002/03 to 2007/08. However, such figures cannot be used to measure prevalence as they can only capture intelligence on individuals admitted to hospital within a given year. Thus, other data collection methods (including clinical and epidemiological studies) produce higher incidence estimates, with worldwide estimates for FAS at 0.97 per 1,000 [1], and more recent estimates for Lazio (Italy) and mixed-racial, mixed socioeconomic populations in the United States of America at up to 7 per 1,000 [32,33]. Nevertheless, our findings highlight the limitations of current recording of FAS and FASD in England, and support the need for further development of the dataset if appropriate services are to be developed. Whilst the levels of alcohol-related harm including attributable hospital admission, mortality and crime have been increasing in recent years [11], no such increases were seen in any of the three diagnoses discussed here: FAS (Q86.0), maternal care for (suspected) damage to the foetus from alcohol (ICD10: O35.4) or foetus and newborn were affected by maternal use of alcohol. Because of the wide age range for children admitted with FAS (Q86.0), interpretation of temporal trends are more complicated for this condition. This is because to some extent the admissions will reflect maternal alcohol consumption patterns up to 15 years earlier, and for which we have no data. We found no evidence of an increase in admissions for FAS among children aged under 1 year, although numbers were admittedly very small, during a period when admissions for alcohol related harms in women of childbearing age increased quite substantially.

We would predict that regional variations in alcohol-related hospital admissions in women of child-bearing age would be related to FASD diagnoses. Thus, it would be expected that the North West and North East regions, known to have higher levels of alcohol misuse and harm (evidenced by hospital admission data presented here as well as other harms including incapacity benefits claimants for alcoholism)[11] would have higher levels of FASD-related conditions. This was not found to be the case, strongly suggesting under-reporting of FASD-related conditions. The argument for under-reporting was strengthened through the outpatient data examined, where no episodes were revealed between 2003/04 and 2007/08 even though children with FASD-related conditions receive treatment as outpatients [3]. However routine outpatient dataset for England cannot provide any intelligence on this at present. Whereas treatment specialty is a mandatory reporting item for outpatient episodes, diagnosis and procedure codes are not [25]. As long as this remains the case, hospitals have little incentive to spend time and resources on reporting them, and hence the information is missing for the majority of records.

To understand the full extent of underreporting, an active ascertainment study is required [29]. The use of alcohol pregnancy screening tools (such as TACE or TWEAK) to identify high risk pregnancies is crucial here [3], although their validation in a UK setting is required. Screening could be performed by nurses, midwives and/or general practitioners, all of whom pregnant women are likely to encounter during their pregnancy. However, exposure to alcohol does not appear to have a direct correlation to outcome [4]. This means that, other than the most extreme cases, risk can be ascribed at birth or during pregnancy but it is not possible to provide a firm diagnosis. This is because difficulties often do not arise until later in life [3]. Without adequate screening or recording, if diagnosis cannot be made until later in life, complications can arise if crucial information has been lost, forgotten or become unavailable. To help address these issues, neonatal discharge summaries could include a section on high risk factors for later disorders including maternal alcohol consumption. This would follow the child and be accessible to them in later life, should such a diagnosis be sought. Finally the methods used to diagnose FASD are not consistent and there appears to be a lack of awareness as to the correct diagnostic framework. This could be addressed through improved training and more consistent use of diagnosis tools (such as the four digit code[3]) by community paediatricians, the most likely professionals to which these children will present to for diagnosis.

## Conclusions

Current intelligence surrounding FASD-related disorders in England is severely limited. Data provided in this report serve to underline the existing gaps rather than present an understanding of the situation. If FASD-related disorders are to be effectively prevented, identified and treated, improvements to intelligence systems, practitioner awareness and screening are essential. It is only by understanding incidence and characteristics of at-risk groups that effective services and interventions can be developed.

## Competing interests

The authors declare that they have no competing interests.

## Authors' contributions

MM and KW developed the idea for the paper. MM, KW and DD wrote the manuscript. DD analysed the data. MM, KW, DD, PAC and MAB contributed to the data analysis. PAC, MAB and RM commented on and contributed to the drafting of the manuscript. MM is the guarantor. All authors read and approved the final manuscript.

## Pre-publication history

The pre-publication history for this paper can be accessed here:

http://www.biomedcentral.com/1471-2431/11/14/prepub
